# M2 macrophage‐derived G‐CSF promotes trophoblasts EMT, invasion and migration via activating PI3K/Akt/Erk1/2 pathway to mediate normal pregnancy

**DOI:** 10.1111/jcmm.16191

**Published:** 2021-01-03

**Authors:** Jinli Ding, Chaogang Yang, Yi Zhang, Jiayu Wang, Sainan Zhang, Duanying Guo, Tailang Yin, Jing Yang

**Affiliations:** ^1^ Reproductive Medical Center Renmin Hospital of Wuhan University & Hubei Clinic Research Center for Assisted Reproductive Technology and Embryonic Development Wuhan China; ^2^ Department of Gastrointestinal Surgery Zhongnan Hospital of Wuhan University Wuhan China; ^3^ Department of Gynecology Longgang District People's Hospital of Shenzhen Shenzhen China

**Keywords:** EMT, G‐CSF, invasion, macrophages, migration, trophoblasts

## Abstract

Trophoblasts are important parts of the placenta and exert vital roles in the maternal‐foetal crosstalk, and sufficient trophoblasts migration and invasion is critical for embryo implantation and normal pregnancy. Macrophages, as the major components of decidual microenvironment at maternal‐foetal interface, can interact with trophoblasts to participate in the regulation of normal pregnancy. Previously, our group have demonstrated that trophoblasts could induce macrophages polarization to M2 subtype by secreting interleukin‐6 (IL‐6); however, the understanding of macrophages regulating the migration and invasion of trophoblasts is limited. In the present study, we used the co‐cultured model to further investigate the effects of macrophages on trophoblasts migration and invasion. Our results showed that co‐culture with macrophages promoted epithelial‐to‐mesenchymal transition (EMT) of trophoblasts, thereby enhancing their migrative and invasive abilities. Further experiments revealed that M2 macrophage‐derived G‐CSF was a key factor, which promoted the EMT, migration and invasion of trophoblasts via activating PI3K/Akt/Erk1/2 signalling pathway. Clinically, G‐CSF was highly expressed in placental villous tissues of normal pregnancy patients compared to patients with recurrent spontaneous abortion, and its expression level was significantly correlation with EMT markers. Taken together, these findings indicate the important role of M2 macrophages in regulating trophoblasts EMT, migration and invasion, contributing to a new insight in concerning the crosstalk between macrophages and trophoblasts in the establishment and maintenance of normal pregnancy.

## 1 INTRODUCTION

Normal pregnancy, as a complex physiological process,^1^ depends not only on the proper maternal‐foetal crosstalk and immune regulation, but also on trophoblast development.^2^ Increasing and accumulating evidence indicates that trophoblast invasion promotes maternal placental blood flow and maternal spiral artery remodelling, thereby establishing favourable microenvironment for embryo implantation.^3^ In contrast, impairment of trophoblast migration and invasion can induce utero‐placental insufficiency, which eventually leads to a series of pregnancy complications, such as foetal growth restriction, pre‐eclampsia and recurrent spontaneous abortion (RSA).^4^, ^5^, ^6^, ^7^ Therefore, exploring the factors affecting trophoblasts invasion and migration is of great significance for further understanding of the normal pregnancy process and the pathogenesis of pregnancy complications. Epithelial‐to‐mesenchymal transition (EMT), as a cellular process in which cells lose their epithelial characteristics and acquire mesenchymal features, has been demonstrated to play a vital role in maintaining the migrative and invasive abilities of trophoblasts in recent years.^8^, ^9^ We and others previously found that inhibition of trophoblasts EMT programme would impair its ability to invade and migrate, resulting in the occurrence of RSA.^7^, ^10^ However, the regulatory mechanisms of trophoblasts EMT still remain elusive.

The initiation and maintenance of EMT status are a multi‐step complex processes cooperating by multiple factors.^9^, ^11^ Emerging studies have demonstrated that intercellular interactions in the maternal‐foetal interface microenvironment play an important role in regulating EMT of trophoblasts.^9^, ^12^ During pregnancy, trophoblasts gain the advantage of invasion and migration by interacting with immune cells, thereby establishing a unique maternal‐foetal microenvironment that contributes to foetal survival and development.^9^, ^13^ Macrophages, as the second largest group of immune cells at the maternal‐foetal interface,^2^ have been demonstrated to play critical roles in embryo implantation, embryonic development, placental formation and delivery processes.^13^, ^14^, ^15^ Previous studies have showed that macrophages can establish ‘crosstalk’ with trophoblasts in the maternal‐foetal interface microenvironment via a complex cytokine‐based connection. On one hand, macrophages can secrete amounts of soluble mediators to regulate the biological behaviours of trophoblasts.^6^, ^13^, ^16^, ^17^, ^18^, ^19^ On the other hand, macrophages can respond to various factors produced by trophoblasts to convert polarization status, thereby playing different biological functions.^20^, ^21^, ^22^ Previously, we have demonstrated that trophoblasts could induce macrophages polarization to M2 subtype by secreting interleukin‐6 (IL‐6), thereby modulating the process of normal pregnancy.^23^ Although the interplay between trophoblasts and macrophages has been established at the maternal‐foetal interface, the understanding of macrophages regulating trophoblasts EMT programme is limited.

In the present study, we utilized an in vitro co‐culture model to investigate the effects of macrophages on EMT, invasion and migration of trophoblasts. The results showed that co‐culture with macrophages promoted EMT programme of trophoblasts, thereby enhancing their migration and migration. Further experiments revealed that M2 macrophage‐derived G‐CSF was a key factor, which promoted the EMT, migration and invasion of trophoblasts via activating PI3K/Akt/Erk1/2 signalling pathway. Clinically, G‐CSF was highly expressed in placental villous tissues of normal pregnancy patients compared to patients with RSA, and its expression level was significantly correlation with EMT markers. Taken together, these findings indicate the important role of M2 macrophages in regulating trophoblasts EMT, migration and invasion, contributing to a new insight in concerning the crosstalk between macrophages and trophoblasts in the establishment and maintenance of normal pregnancy.

## 2 MATERIALS AND METHODS

### 2.1 Patients and tissue samples

Women conducted induced abortion for non‐medical reasons were chose for control group, and patients suffered from recurrent spontaneous miscarriage for two or more times were regarded as RSA group. Twenty‐one RSA patients and 28 normal pregnant women from Renmin Hospital of Wuhan University were included in the study. RSA patients with characteristics including endocrine or metabolic diseases (such as diabetes, hyperthyroidism, hypothyroidism), uterine abnormality, abnormal karyotype or infection (according to the leucorrhoea routine examination) were excluded from the study. The placental villous tissues and decidual tissue samples were collected at the time of surgery, fixed in 4% paraformaldehyde for paraffin‐embedding in blocks or frozen and stored in liquid nitrogen. The procedure was performed with the approval of the internal review and ethics boards of Renmin Hospital of Wuhan University, and informed consent was obtained from all included patients. The baseline characteristics of the patients were summarized in Table 1.

**TABLE 1:** Baseline clinical characteristics of the study population

Parameters	RSA	Normal pregnancy
Maternal age (year)	32.3 ± 2.78	30.78 ± 3.29
Body mass index (kg/m^2^)	21.56 ± 1.76	22.13 ± 1.34
Gestation age (weeks)	8.56 ± 1.38	8.34 ± 1.25
Number of pregnancies	2.43 ± 0.32	1.86 ± 0.34
Number of live births	0.00 ± 0.00	1.45 ± 0.32

Abbreviation: RSA, recurrent spontaneous abortion.

### 2.2 Cell culture and reagents

The human monocyte cell line THP‐1 was cultured and grown in RPMI‐1640 medium (Gibco, USA) with 10% foetal bovine serum (FBS) (Gibco, USA), and the trophoblast cell line, HTR‐8/SVneo (HTR‐8), was grown in DMEM‐F12 medium (Gibco, USA) with 10% FBS, at 37°C in the presence of 5% CO_2_. For macrophage generation, THP‐1 cells were treated with 50 ng/ml Phorbol 12‐myristate 13‐acetate (PMA; Sigma, USA) for 24 h. Macrophages and trophoblasts co‐cultivation was conducted using the non‐contact co‐culture transwell system (Corning, USA) for 72h, in which macrophages were seeded in 0.4 μm sized pores inserts, and HTR‐8 was seeded in the 6‐well plate (1.5 × 10^5^ cells per well).

Recombinant human G‐CSF (Proteintech, USA) was dissolved in ddH_2_O and used at a final concentration of 100 ng/mL. LY294002 (PI3K inhibitors) and PD 98059 (Erk1/2 inhibitors) were purchased from Med Chem Express, China. The anti‐human neutralizing G‐CSF antibody was acquired from R&D Systems, USA.

### 2.3 RNA isolation and quantitative real‐time PCR (RT‐PCR)

The total RNA was isolated using the TRIzol Reagent (Invitrogen, USA) according to the manufacturer's instructions. After detection of RNA concentration, 0.5 μg of total RNA was reverse‐transcribed into cDNA using the RT reagent kit (Taraka, Japan), and the cDNA was used for subsequent RT‐PCR using the SYBR‐Green PCR Mix (Takara) by a 7500 Real‐Time PCR (Applied Biosystems, MA, USA). The relative mRNA expression level was calculated using the 2^‐ΔΔCt^ method. The sequences of primers for RT‐PCR were as following: E‐cadherin, Forward: 5′‐ATTTTTCCCTCGACACCCGAT‐3′, Reverse: 5′‐TCCCAGGCGTAGACCAAGA‐3′; Vimentin, Forward: 5′‐AGTCCACTGAGTACCGGAGAC‐3′, Reverse: 5′‐CATTTCACGCATCTGGCGTTC‐3′; G‐CSF, Forward: 5′‐ATAGCGGCCTTTTCCTCTACC‐3′, Reverse: 5′‐GCCATTCCCAGTTCTTCCAT‐3′; GAPDH, Forward: 5′‐CACTGGGCTACACTGAGCAC‐3′, Reverse: 5′‐AGTGGTCGTTGAGGGCAAT‐3′.

### 2.4 Western blot

Protein extraction and Western blot were performed as we previously reported.^7^ The following primary antibodies were used: anti‐E‐cadherin (1:1000; Proteintech, USA), anti‐Vimentin (1:1000; Proteintech), anti‐p‐Erk1/2 (phosphor T202 + T204) (1:1000; Cell Signaling Technology, CST, USA), anti‐Erk1/2 (1:1000; CST), anti‐p‐Stat3 (phosphor Y705) (1:1000; CST), anti‐Stat3 (1:1000; CST), anti‐p‐Akt (phosphor S473) (1:1000; CST), anti‐Akt (1:1000; CST), anti‐p‐P38 (1:1000; CST), anti‐P38 (1:1000; CST) and anti‐GAPDH (1:5000; Proteintech).

### 2.5 Cytokine assays

The cytokine antibody array agented by Wayen Biotechnologies (Shanghai, China) was used to detect inflammatory cytokines in the supernatant obtained from HTR‐8, macrophages and the co‐culture model, which contains cytokines associated with invasion including MCP‐1, IP‐10, IL‐6, VEGF, G‐CSF, IL‐15, IL‐1β, IL‐10 and IL‐12. Results were expressed as pictograms per millilitre.

### 2.6 Wound healing assay

The wound healing assay was used to evaluate the migration ability of trophoblasts. A wound was made by dragging the plastic pipette tip across the cell surface when cells were grown to 80% confluence in 24‐well plates, and migrating cells at the wound front were photographed after 24h and 48h. The area of the wound was measured with Image J software (NIH, USA).

### 2.7 Transwell invasion assay

Cell invasion assays were performed using 24‐well transwells (8µm pore size; Corning, USA) and coated with Matrigel (Corning, USA). In total, cells suspended in 500 µl DMEM/F‐12 were added to the upper chamber, while 500 µl DMEM/F‐12 containing 5% FBS was placed in the lower chamber. After 48h of incubation, Matrigel and the cells remaining in the upper chamber were removed using cotton swabs. Cells on the lower surface of the membrane were stained with 0.5% crystal violet for 15 min. Cells from five fields at a magnification of × 200 were counted. All experiments were performed in triplicate.

### 2.8 Immunohistochemistry (IHC)

For IHC, serial sections from the human placental villous tissues and the decidual tissues were obtained. IHC was conducted as previously described.^24^ The following primary antibodies were used: anti‐CD163 (Proteintech), anti‐Vimentin (Proteintech), anti‐G‐CSF (Abcam, USA) and anti‐E‐cadherin (Proteintech). Five visual fields were selected and observed in each section, and cells counted at 200 × magnification in each case using average values.

### 2.9 Statistics analysis

All statistical analyses were performed using SPSS 22. 0 (SPSS Statistics, IL, USA) and GraphPad Prism 6.0 (GraphPad Software Inc, CA, USA) for Windows. Groups of discrete variables were compared by means of the Kruskal‐Wallis non‐parametric analysis of variance or Mann‐Whitney *U* test. Pearson correlation was used to analysis the correlations between the variables in the decidual and placental villous tissues. *P* values < 0.05 were considered statistically significant.

## 3 RESULTS

### 3.1 Co‐culture with macrophages promote EMT, migration and invasion of trophoblasts

**FIGURE 1:**
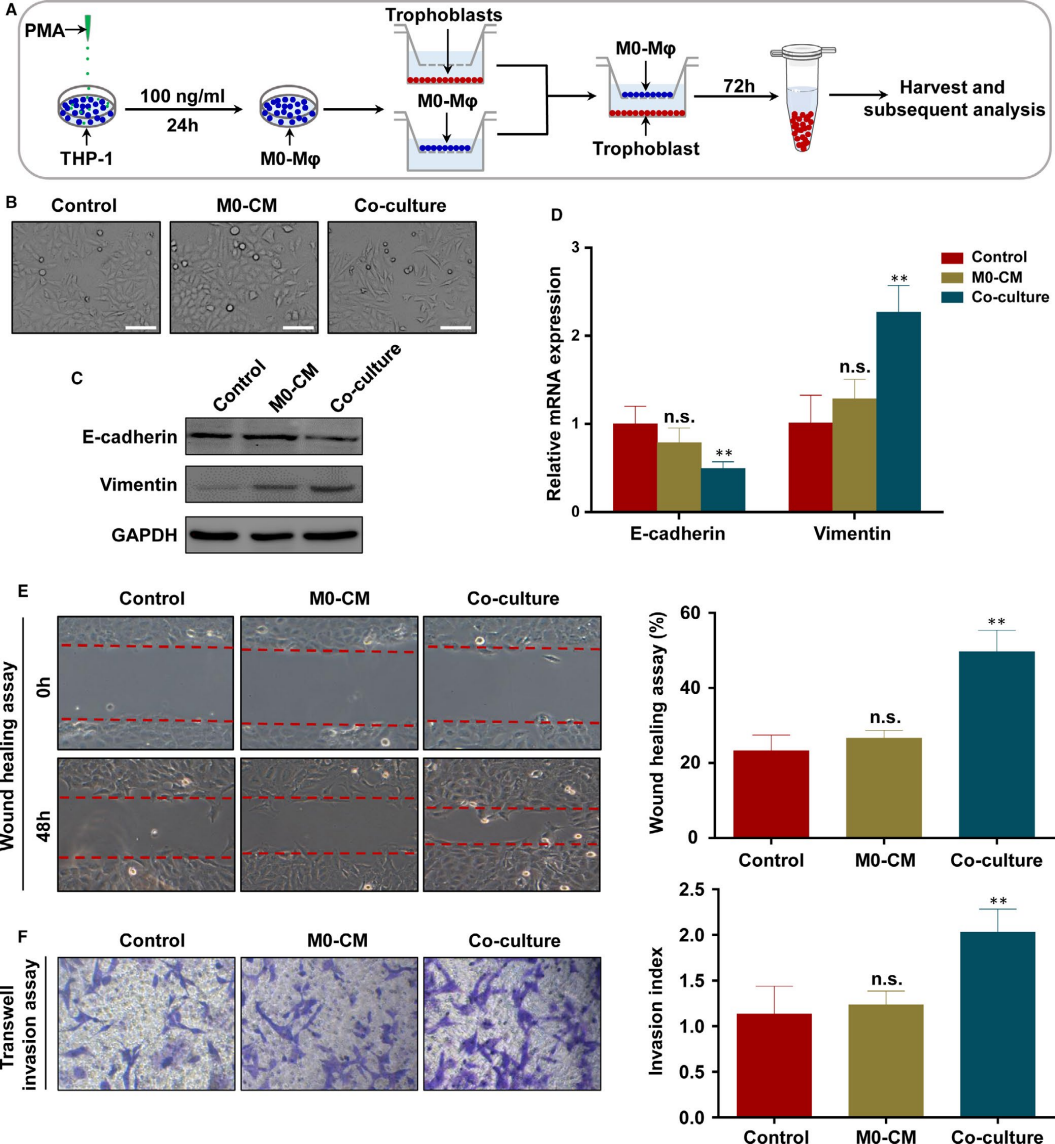
Co‐culture with macrophages promote EMT, migration and invasion of trophoblasts. A, Schema of THP‐1‐derived macrophage‐HTR‐8 co‐culture model. B, HTR‐8 was cultured with PRIM‐1640, M0 macrophage‐conditioned media or co‐cultured with M0 macrophages for 72 h. The representative bright‐field images of HTR‐8 are shown. Scale bar, 50 μm. C, The effect of the co‐culture model on the EMT of HTR‐8 was analysed by Western blot analysis. The results were representative of three separate experiments. D, RT‐PCR assays of E‐cadherin and Vimentin in HTR‐8 grown in PRIM‐1640, M0 macrophage‐conditioned media or co‐cultured with macrophages for 72h. E and F, Cell migration and invasion capacity of HTR‐8 grown in PRIM‐1640, M0 macrophage‐conditioned media or co‐cultured with macrophages was determined by wound healing assay and transwell system, respectively. Representative photographs of migratory or invasive cells (magnification, ×200) are shown. Notes: ^**^ *P* < .01; n.s., not significant

To determine the effect of macrophages on trophoblasts, we utilized an in vitro co‐culture model using the trophoblast cell line HTR‐8 and PMA‐treated THP‐1 macrophages (M0‐Mφ), which were impermeable for cells and allowed the exchange of soluble factors between two cells (Figure 1A). HTR‐8 treated with the culture medium of PMA‐treated THP‐1 macrophages (M0‐CM) or PBS was regarded as control. Macrophages co‐culture led to a spindle‐shaped morphology and increased the formation of pseudopodia in HTR‐8, compared with the control and M0‐CM group (Figure 1B). To investigate whether macrophages could induce EMT of trophoblasts in vitro, Western blot and RT‐PCR were performed to analyse the EMT markers in HTR‐8 co‐cultured with M0‐Mφ for 72h. As shown in Figure 1C‐D, the expression of epithelial marker E‐cadherin was reduced, while the mesenchymal marker Vimentin was up‐regulated in the co‐culture model, while there was no significant difference in the expression of E‐cadherin or Vimentin between M0‐CM and control group. Meanwhile, to further verify whether macrophages directly induced the migration and invasion of HTR‐8, wound healing assay and transwell invasion assays were conducted. The results showed that HTR‐8 co‐cultured with macrophages exhibited a faster closure of the wound when compare to control group (Figure 1E), and similar results were obtained in transwell assay (Figure 1F), while M0‐CM had no obvious effect. In addition, we previously investigated the phenotype of macrophages in above co‐culture model, and the results showed that trophoblasts could secret interleukin‐6 (IL‐6) to induce macrophages polarization to M2 subtype.^23^ Combine our previous and present results, we conclude that macrophages mainly exhibit M2 subtype in this co‐culture model in vitro, which promotes the EMT programme of trophoblasts to enhance their migrative and invasive abilities.

### 3.2 G‐CSF is newly identified and validated as a key factor for M2 macrophage‐induced trophoblasts EMT, migration and invasion

**FIGURE 2:**
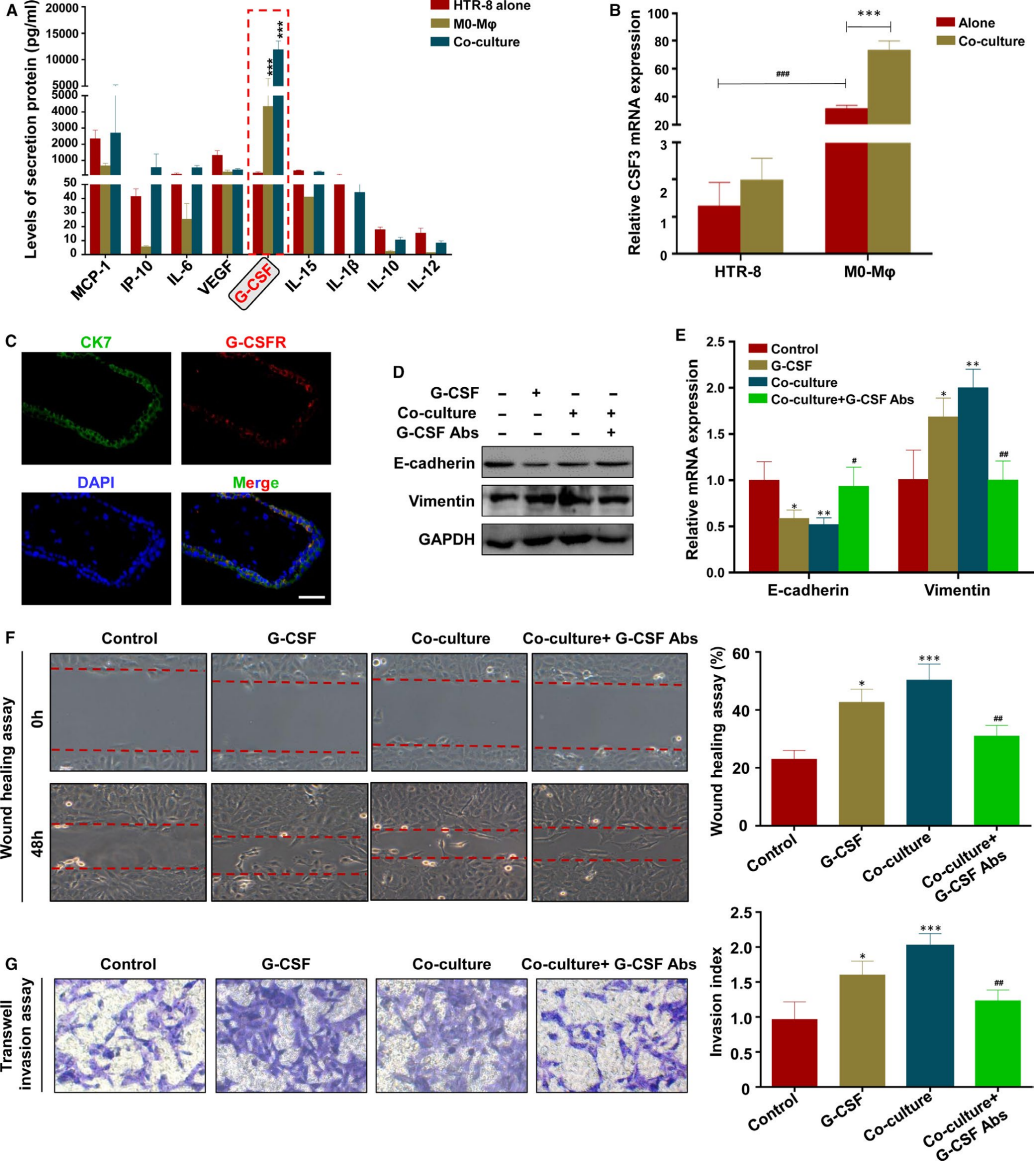
G‐CSF is newly identified and validated as a key factor for M2 macrophage‐induced trophoblasts EMT, migration and invasion. A, The cytokines assay of indicated molecules in the culture medium of HTR‐8, TEMs or the co‐culture model was conducted with cytokine antibody array agented by Wayen Biotechnologies. B, The mRNA expression of CSF3 in HTR‐8 and M0 macrophages with or without 72h of co‐culture were determined by RT‐PCR. C, Immunostaining for G‐CSFR in CK7^+^ trophoblasts (Scale bar = 10 μm). D and E, Expression of E‐cadherin and Vimentin in HTR‐8 alone, G‐CSF‐stimulated HTR‐8, macrophage‐co‐cultured HTR‐8 and G‐CSF depleted macrophage‐co‐cultured HTR‐8 were analysed by Western blot and RT‐PCR, respectively. F and G, Cell migration and invasion capacity in HTR‐8 alone, G‐CSF‐stimulated HTR‐8, macrophage‐co‐cultured HTR‐8 and G‐CSF depleted macrophage‐co‐cultured HTR‐8 were determined by wound healing assay and transwell system, respectively. Representative photographs of migratory or invasive cells (magnification, ×200) are shown. Notes: ^*^ *P* < .05, ^**^ *P* < .01, ^***^ *P* < .001, compared with control group; ^#^ *P* < .05, ^##^ *P* < .01, compared with co‐culture group

Previous studies have indicated that macrophages exerted their ability on invasion and migration through the secretion of paracrine factors,^13^, ^14^, ^15^ and we therefore examined the factors secreted by macrophages which might play a regulating role on the invasive and migratory activities of trophoblasts. The cytokines assay showed that the level of granulocyte colony‐stimulating factor (G‐CSF) emerged as the most prominently up‐regulated cytokine in co‐cultured model than those in HTR‐8 or M0‐Mφ (Figure 2A). RT‐PCR further showed the basal level of CSF3 mRNA was much higher in macrophages than HTR‐8, and the co‐cultured model promoted CSF3 mRNA expression in macrophages but not in HTR‐8 (Figure 2B), suggesting that most of the CSF3 was derived from macrophages, and macrophages might modulate the migration and invasion of HTR‐8 in the co‐culture model via G‐CSF. In addition, we detected the G‐CSF receptor (G‐CSFR) on trophoblasts in the placental villous tissues and found the co‐localization of G‐CSFR and Cytokeratin 7 (CK7) (Figure 2C). To evaluate whether G‐CSF was critical for the EMT process of HTR‐8, recombinant G‐CSF was added in the culture medium of HTR‐8. The results showed that G‐CSF significantly reduced the expression of E‐cadherin, while increased the expression of Vimentin in both protein and mRNA level (Figure 2D‐E).

Furthermore, a G‐CSF neutralizing antibody was used to confirm the regulatory effect of G‐CSF in HTR‐8. After adding the G‐CSF neutralizing antibody into the co‐culture model, the expression of Vimentin was decreased, while E‐cadherin was increased when compared with G‐CSF treatment group (Figure 2D‐E). Consistently, the G‐CSF neutralizing antibody decreased the migratory (Figure 2F) and invasive capacities (Figure 2G) of HTR‐8. Collectively, these findings indicate that M2 macrophage‐derived G‐CSF is newly identified and validated as a key factor for M2 macrophage‐induced trophoblasts EMT.

### 3.3 M2 Macrophage‐derived G‐CSF promotes EMT, migration and invasion of trophoblasts via activating PI3K/Akt/Erk1/2 pathway

**FIGURE 3:**
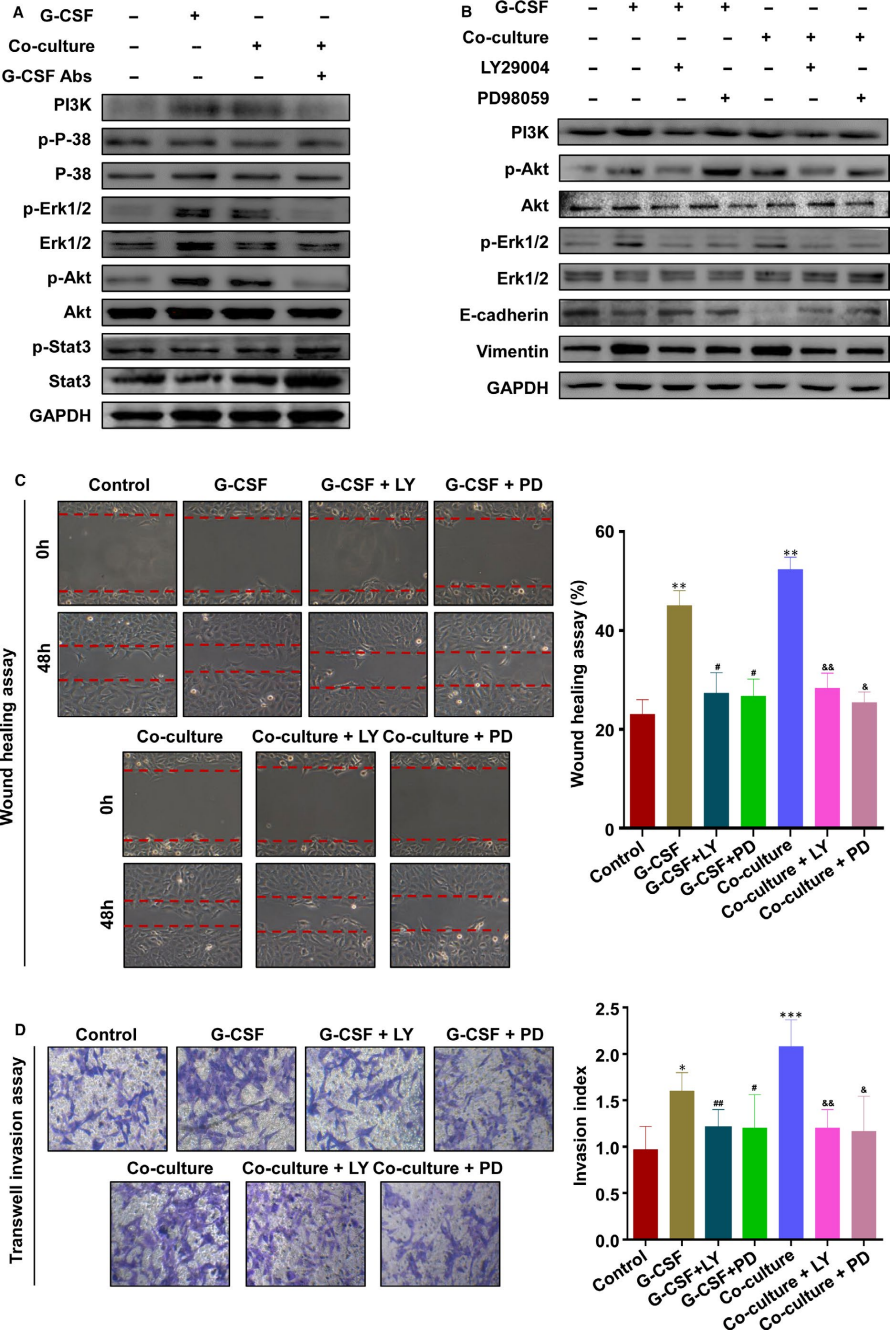
M2 Macrophage‐derived G‐CSF promotes EMT, migration and invasion of trophoblasts via activating PI3K/Akt/Erk1/2 pathway. A, Western blot analysis of HTR‐8 alone, G‐CSF‐stimulated HTR‐8, macrophage‐co‐cultured HTR‐8 and G‐CSF depleted macrophage‐co‐cultured HTR‐8. B, Western blot analysis of HTR‐8 alone, G‐CSF‐stimulated HTR‐8 and macrophage‐co‐cultured HTR‐8 in the presence or absence of LY29004 (20 μM) or PD98059 (15 μM). C and D, Cell migration and invasion capacity in HTR‐8 alone, G‐CSF‐stimulated HTR‐8 and macrophage‐co‐cultured HTR‐8 in the presence or absence of LY29004 (20 μM) or PD98059 (15 μM) were determined by wound healing assay and transwell system, respectively. Representative photographs of migratory or invasive cells (magnification, ×200) are shown. Notes: compared with control group, ^*^ *P* < .05, ^**^ *P* < .01, ^***^ *P* < .001; compared with G‐CSF‐stimulated HTR‐8 group, ^#^ *P* < .05, ^##^ *P* < .01; compared with co‐culture group, ^&^ *P* < .05, ^&&^ *P* < .01

To determine which downstream signals in HTR‐8 responded to G‐CSF secretion by M2 macrophages, we focused on PI3K, Erk1/2, Akt and P‐38 pathway, which have been reported to be activated in trophoblasts treated with G‐CSF.^25^, ^26^, ^27^, ^28^ The results found that the stimulation of HTR‐8 with G‐CSF or co‐cultured with macrophages increased the expressions of PI3K, p‐Akt and p‐Erk1/2, whereas the treatment of G‐CSF neutralizing antibody inhibited co‐cultured‐induced expressions of PI3K, p‐Akt and p‐Erk1/2 (Figure 3A). To investigate the role of PI3K, Akt and Erk1/2 signalling in macrophage‐induced EMT, treatment of LY294002 (PI3K inhibitors) or PD98059 (Erk1/2 inhibitors) was used in the next experiments. The results showed that LY294002 markedly blocked G‐CSF or co‐cultured‐induced expressions of PI3K, p‐Akt and p‐Erk1/2 (Figure 3B), and PD98059 markedly blocked G‐CSF or co‐cultured‐induced expressions of p‐Erk1/2, without obvious effect on the expression of PI3K and p‐Akt (Figure 3B). In addition, both of LY294002 and PD98059 attenuated the promoting role of G‐CSF or co‐culture on the expression of Vimentin and also the inhibitory effect on E‐cadherin (Figure 3B). Moreover, the wound healing assay showed that in the presence of the Erk1/2 inhibitor or PI3K inhibitors, the gap closure was significantly reduced, compared with treatment of G‐CSF or co‐culture model (Figure 3C). Similar results were observed in invasion assay (Figure 3D). These data demonstrate that M2 macrophage‐derived G‐CSF promotes EMT, migration and invasion of trophoblasts via activating PI3K/Akt/Erk1/2 pathway.

### 3.4 High expression of G‐CSF in placental villous tissues is associated with normal pregnancy

**FIGURE 4:**
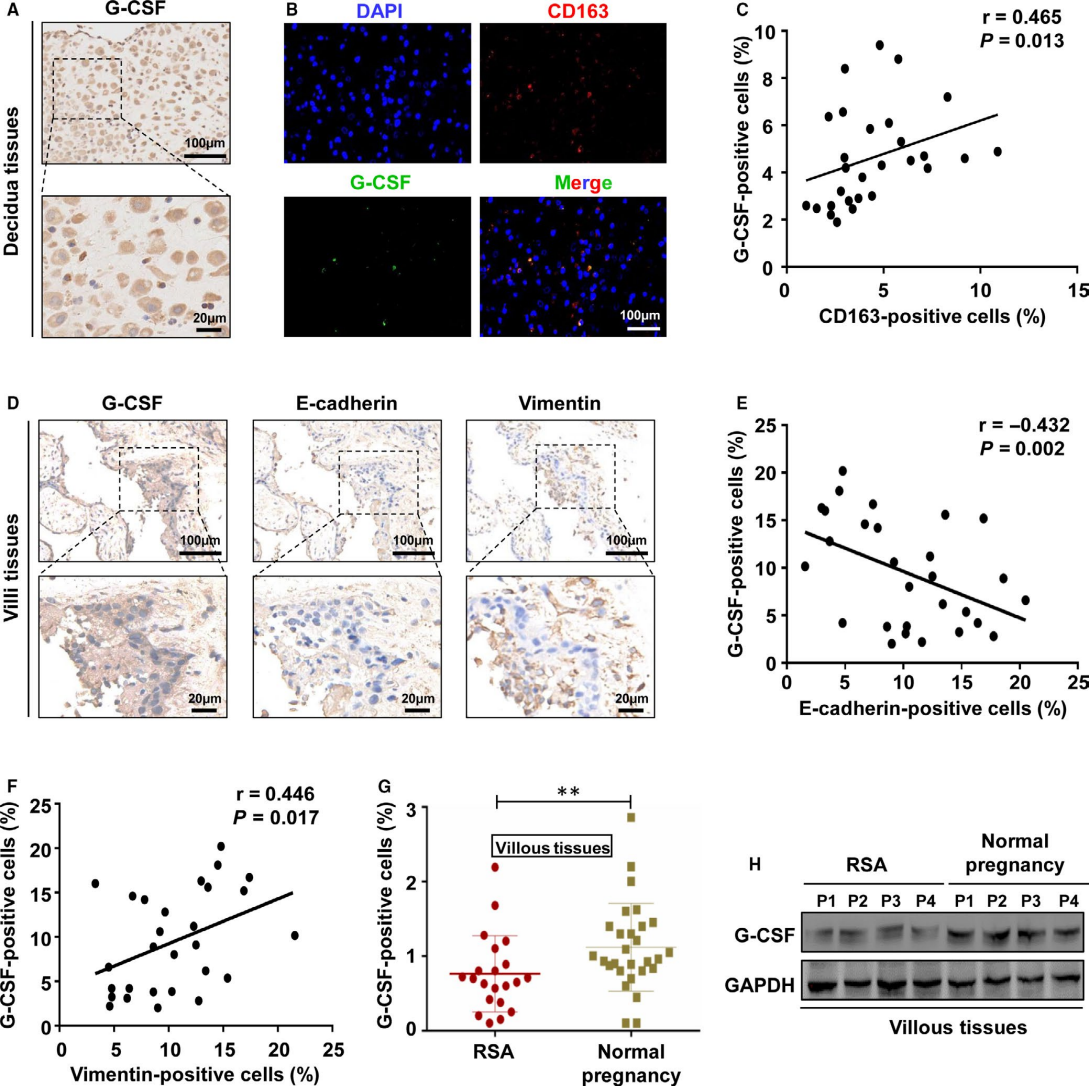
High expression of G‐CSF in placental villous tissues is associated with normal pregnancy. A, The distribution of G‐CSF in the decidual tissues of normal pregnancy was detected by IHC. Scale bars, 100 μm and 20 μm. B, The distribution of G‐CSF on CD163^+^ macrophages was analysed by immunofluorescence. Scale bar, 100 μm. C, Positive correlation between levels of G‐CSF and CD163 in the decidual tissues of normal pregnancy (n = 28; r = 0.465, *P* = .013). Scale bars, 100 μm and 20 μm. D, IHC analysed the expression of G‐CSF, E‐cadherin and Vimentin protein in the placental villous tissues of normal pregnancy. E, Negative correlation between levels of G‐CSF and E‐cadherin in the placental villous tissues of normal pregnancy (n = 28; r=−0.432, *P* = .002). F, Positive correlation between levels of G‐CSF and Vimentin in the placental villous tissues of normal pregnancy (n = 28; r = 0.446, *P* = .017). G and H, The mRNA expression levels of G‐CSF and protein levels of G‐CSF were analysed with RT‐PCR and Western blot, respectively. Note: ^**^ *P* < .01

Furthermore, we evaluated the expression of G‐CSF in the placental villous and decidual tissues from normal pregnancy. The results found that G‐CSF was expressed on decidual stromal cells, with membrane patterns (Figure 4A). To confirm the connection between G‐CSF and M2 macrophages in the decidua, we investigated the expressions of G‐CSF and CD163 by double immunofluorescence staining. As shown in Figure 4B, the results showed prolific G‐CSF protein on CD163^+^ macrophages. Further analysis indicated that the proportion of G‐CSF^+^CD163^+^ macrophages in CD163^+^ macrophages was 52.24%±8.76%, and the proportion of G‐CSF^+^CD163^+^ and G‐CSF^+^CD163^‐^ in the decidua were 6.78%±3.56% and 15.26%±6.82%, respectively. In addition, a positive correlation was found between the proportion of CD163^+^ and G‐CSF^+^ cells (r = 0.465, *P* = .013; Figure 4C). Then, IHC was performed to analysis the expression of G‐CSF, E‐cadherin and Vimentin in the placental villous tissues in serial sections. The results demonstrated that the expression of G‐CSF was observed in placental villous tissues, and the G‐CSF^+^ cells were mainly located on cytotrophoblasts, a few on syncytiotrophoblasts (Figure 4D). Furthermore, high level of G‐CSF was associated with less E‐cadherin and more Vimentin (Figure 4D). Pearson test showed that there was a statistically significant negative correlation between the expression levels of G‐CSF and E‐cadherin (r=−0.432, *P* = .002; Figure 4E), while a significant positive correlation between the expression levels of G‐CSF and Vimentin in the placental villous tissues (r = 0.446, *P* = .017; Figure 4F). To further explore the role of G‐CSF in pregnancy, we analysed the expression of G‐CSF in the placental villous tissues from women with normal pregnancy and patients suffered from RSA by RT‐PCR and Western blot. The results found that G‐CSF mRNA and protein expression were highly expressed in the placental villous tissues of normal pregnancy patients, compared with patients with RSA (Figure 4G‐H). Taken together, these findings indicate that high expression of G‐CSF in placental villous tissues is associated with normal pregnancy.

**FIGURE 5:**
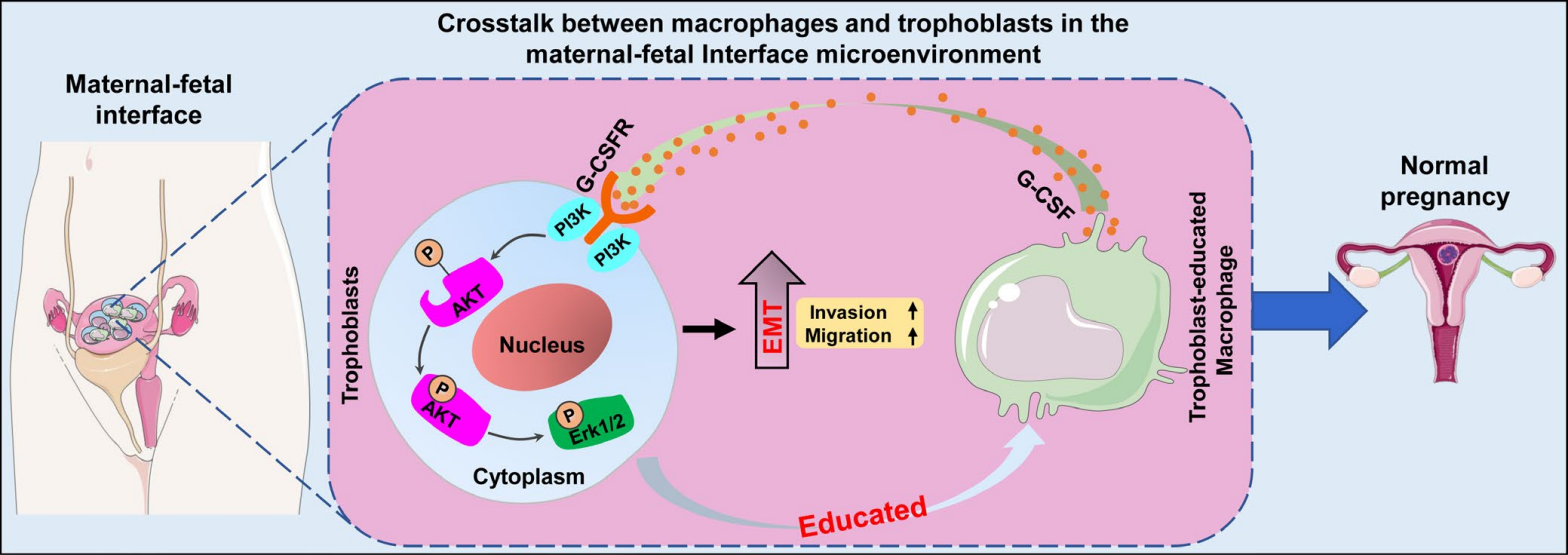
Schema of the interaction between trophoblasts and macrophages at the maternal‐foetal interface. Our study illustrated G‐CSF, secreted by trophoblast‐educated M2 macrophages, binds to the G‐CSF receptor (G‐CSFR) on trophoblasts surface to phosphorylate PI3K/Akt/Erk1/2 signalling pathways, which facilitates the EMT programme to enhance the migration and invasion of trophoblasts, thereby participating in regulating the establishment and maintenance of normal pregnancy

We summarized our findings in a schematic (Figure 5). Our study illustrated G‐CSF, partially secreted by trophoblast‐educated M2 macrophages, could bind to the G‐CSF receptor (G‐CSFR) on trophoblasts surface to phosphorylate PI3K/Akt/Erk1/2 signalling pathways, which facilitates the EMT programme to enhance the migration and invasion of trophoblasts, thereby participating in regulating the establishment and maintenance of normal pregnancy.

## 4 DISCUSSION

Successful pregnancy is a complex process involving multiple cells and factors.^1^ Macrophages, as one of the main immune cells in the maternal‐foetal interface, have been shown to interact with trophoblasts, the main component of placental villous tissues, to play a role in the normal development of the placenta and the process of embryo implantation.^2^ Therefore, exploring the interaction of macrophages and trophoblasts is important for understanding the physiological processes of normal pregnancy. In the present study, we demonstrated that trophoblast‐educated M2 macrophages could regulate the migration and invasiveness of trophoblasts through PI3K/Akt/Erk1/2 signalling pathways, via the soluble factor G‐CSF, and the expression of G‐CSF at the maternal‐foetal interface was associated with M2 macrophages. In addition, high expression of G‐CSF in placental villous tissues is associated with normal pregnancy, which confirming the importance of crosstalk of macrophages and trophoblasts and G‐CSF in the process for the establishment of normal gestation.

Macrophages account for approximately 20%‐30% decidual leucocytes, having marked influence on the local environment and thus EVTs function.^29^ In fact, the accumulation of macrophages at the implantation site and the invasive front of EVTs has been proven.^30^ Paracrine activity of macrophages exerts indispensable roles in EVTs formation, and a range of factors secreted by decidual macrophages has been shown to alter EVTs motility.^15^ A previous study from rhesus monkey demonstrated that macrophages had regulatory effects on the growth and differentiation of embryonic trophoblasts.^31^ In addition, researches also indicated that macrophages with different polarization states had different regulatory effects on the motility and tube formation of trophoblasts.^19^ Conversely, inhibitory/regulatory activation characteristics and cytokine expression of macrophages could be induced by trophoblasts.^21^, ^32^ Our present study further deepened the understanding of the interaction between trophoblasts and macrophages, which was, macrophages educated by trophoblasts could in turn promoted the invasive and migratory ability of trophoblasts.

Given the important roles of cytokines in cell‐cell interactions, we screened the changes of a panel of cytokines in the co‐cultured model, and G‐CSF was confirmed as the most significantly up‐regulated cytokine. Subsequent functional assays identified that G‐CSF was involved in macrophage‐induced EMT, migration and invasion of trophoblasts. Previous study from other trophoblast cell lines including Swan 71 cell^26^, ^27^ and JEG‐3 cell^28^ have proven the role of G‐CSF on the migration and invasion of trophoblasts, which was partially strengthen by our results. G‐CSF belongs to the family of CSF, synthesized by multiple cell types including fibroblasts, endothelial cells, lymphocytes and macrophages.^33^, ^34^ Shorter *et al* first reported that placental villous and decidual tissues were sources of G‐CSF in 1992,^35^ which was confirmed by Miyama *et al* that G‐CSF was secreted by decidual cells and decidual macrophages were sources of G‐CSF in 1998.^36^ Numerous researches have confirmed the role of G‐CSF in pregnancy. G‐CSF has been regarded the biomarker of oocyte quality and implantation potential, as follicular G‐CSF was associated with the number of fertilized oocytes.^37^, ^38^ A positive relationship was reported that high concentrations of G‐CSF in follicular fluid and the probability of implantation and subsequent pregnancy.^39^ As the effectiveness of G‐CSF has been demonstrated in improvement in oocyte quality, ovarian follicular function,^40^ endometrial thickness, blood supply^41^ and trophoblast function,^26^, ^27^, ^28^ increasing researches have reported the clinical application of G‐CSF in thin endometrium, luteinized unruptured follicle syndrome, repeated implantation failure and RSA,^34^, ^41^, ^42^, ^43^ although the effectiveness is controversial. A body of growing evidence has confirmed the roles of G‐CSF on the trophoblast function, including the migrating capability,^27^ MMP‐2 activity and VEGF secretion^26^ and survival^28^; however, the mechanism was not clear. G‐CSF was involved in several physiological and pathological processes by complicated mechanisms, such as activation of MAPK and JAK2/STAT3 pathway.^28^, ^44^, ^45^ Moreover, it has been reported that G‐CSF could increase migration of Swan71 cell via PI3K and MAPK activation.^27^ In the present study, our results demonstrated that trophoblast‐educated M2 macrophages promoted the EMT process, migration and invasion of trophoblasts via PI3K/Akt/Erk1/2 signal pathway. In addition, we observed a positive correlation between the expression of G‐CSF and the percentage of CD163^+^ cells, which was in accordance with the result that high levels of G‐CSF were associated with increased CD163^+^ macrophages in triple‐negative breast cancer.^46^ G‐CSF has been regarded as a M2 marker,^47^ and our previous study has confirmed that trophoblasts educated macrophages to M2 subtype,^23^ so we considered that trophoblast‐educated M2 macrophages secreted G‐CSF to promote the invasion and migration of trophoblasts. In addition, a considerable number of G‐CSF^+^CD163^‐^ cells were found in the decidua, indicating that in addition to macrophages, other cells in the decidua, such as NK cells, were also sources of G‐CSF.^48^ The present study demonstrated high expression of G‐CSF in placental villous tissues is associated with normal pregnancy, which highlights the importance of macrophages and trophoblasts crosstalk at the maternal‐foetal interface. Given that the role of insufficient invasion of trophoblasts and decreased M2 macrophages in RSA has been confirmed by others’ and our previous study,^6^, ^7^, ^17^, ^49^ we could speculate that decreased proportion of M2 macrophages reduces the level of G‐CSF to a certain extent and then participates in the occurrence of RSA by affecting the invasion and migration of trophoblasts. However, the lower G‐CSF in the placental villous tissues of RSA might be a consequence of miscarriage, as the pregnancy might have failed some time before the sample collection, which might be partially verified by animal experiments.

## 5 CONCLUSION

In summary, our current findings disclose the important role of macrophages in regulating the EMT programme, migration and invasion of trophoblasts, contributing to a new insight concerning the crosstalk between macrophages and trophoblasts in the establishment and maintenance of normal pregnancy.

## CONFLICT OF INTEREST

The authors declare that there is no conflict of interest.

## AUTHORS’ CONTRIBUTIONS

Jing Yang, Tailang Ying and Duanying Guo designed the research. Jinli Ding, Chaogang Yang and Yi Zhang performed the experiments and analysed the data. Jinli Ding, Jiayu Wang and Sainan Zhang collected the clinical samples and patients’ data. Jinli Ding, Chaogang Yang and Yi Zhang wrote the draft. Jing Yang, Tailang Ying and Duanying Guo reviewed and revised the manuscript. All authors read and approved the final manuscript.

## Data Availability

All data generated or analysed during this study are included in this published article.
