# Corrigendum

**DOI:** 10.1111/jcmm.17501

**Published:** 2022-09-05

In Jinli Ding et al.,^1^ the published article contains errors in Figure 3C, D. The wound healing picture from the group of Co‐culture + PD in Figure 3C and the image of invasion capacity for G‐CSF + LY in Figure 3D are incorrect in the original publication due to technical error during image preparation. The corrected Figure 3 is shown below. The authors confirm that all the results and conclusions of this article remain unchanged.

**FIGURE 3:**
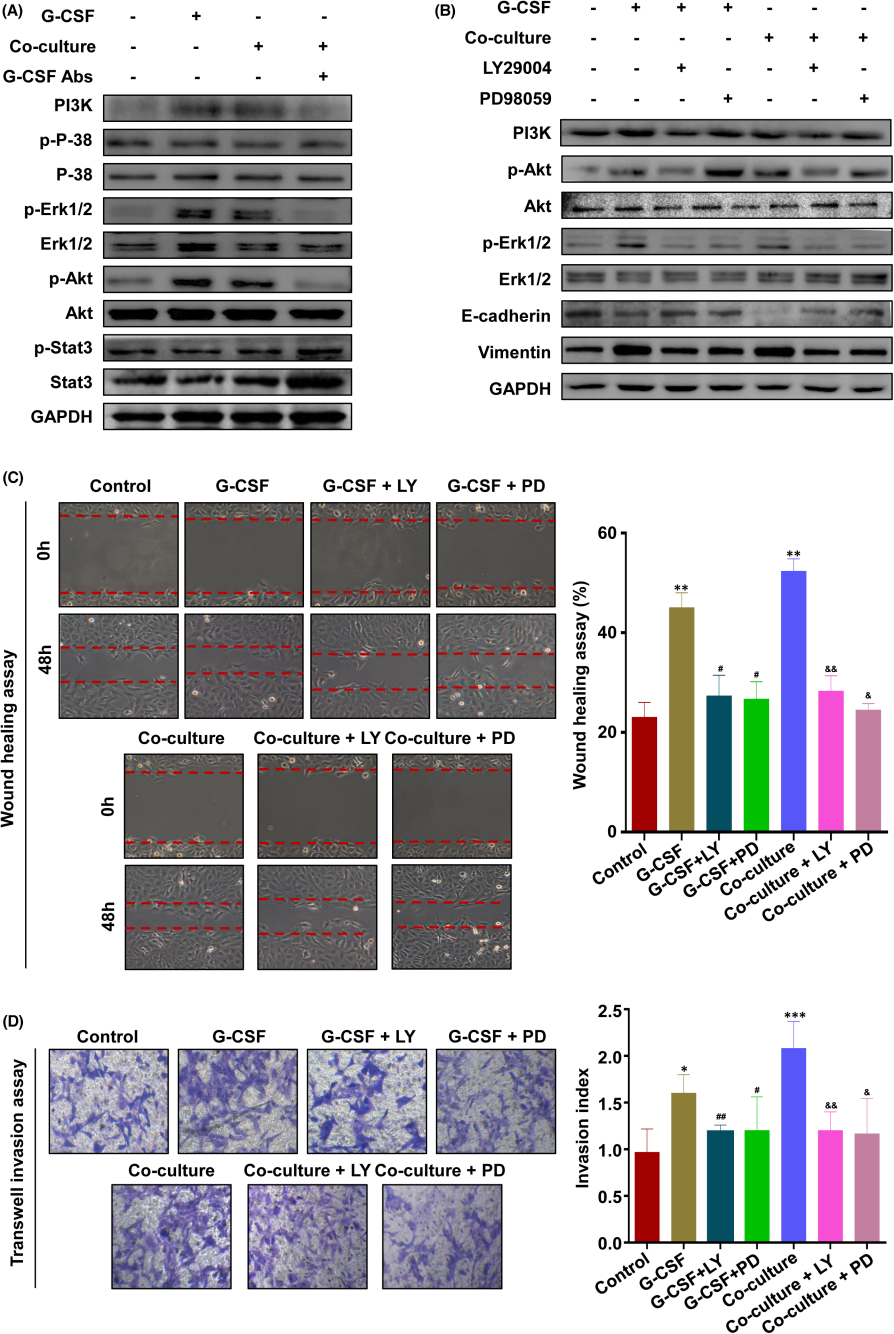
M2 macrophage‐derived G‐CSF promotes EMT, migration and invasion of trophoblasts via activating PI3K/Akt/Erk1/2 pathway. A, Western blot analysis of HTR‐8 alone, G‐CSF‐stimulated HTR‐8, macrophage‐co‐cultured HTR‐8 and G‐CSF depleted macrophage‐co‐cultured HTR‐8. B, Western blot analysis of HTR‐8 alone, G‐CSF‐stimulated HTR‐8 and macrophage‐co‐cultured HTR‐8 in the presence or absence of LY29004 (20 μM) or PD98059 (15 μM). C and D, Cell migration and invasion capacity in HTR‐8 alone, G‐CSF‐stimulated HTR‐8 and macrophage‐co‐cultured HTR‐8 in the presence or absence of LY29004 (20 μM) or PD98059 (15 μM) were determined by wound healing assay and transwell system, respectively. Representative photographs of migratory or invasive cells (magnification, ×200) are shown. Notes: compared with control group, *p < 0.05, **p < 0.01, ***p < 0.001; compared with G‐CSF‐stimulated HTR‐8 group, #*p* < 0.05, ##*p* < 0.01; compared with co‐culture group, &*p* < 0.05, &&*p* < 0.01
